# Prevalence and feasibility of assessing the frailty phenotype among hemodialysis patients in a dialysis unit

**DOI:** 10.1186/s12882-023-03413-w

**Published:** 2023-12-13

**Authors:** Anuradha Wadhwa, Salva N. Balbale, Sujith K. Palleti, Manpreet Samra, Reynold I. Lopez-Soler, Kevin T. Stroupe, Talar W. Markossian, Megan Huisingh-Scheetz

**Affiliations:** 1 Department of Medicine/ Nephrology, Edward Hines Jr. Veterans Administration Hospital, Hines, IL USA; 2 Department of Medicine/ Nephrology, Loyola University Chicago, Maywood, IL USA; 3 Division of Gastroenterology and Hepatology, Department of Medicine, Center for Health Services and Outcomes Research, Institute of Public Health and Medicine, Northwestern Quality Improvement, Research, & Education in Surgery (NQUIRES), Department of Surgery, Northwestern University Feinberg School of Medicine, Chicago, IL USA; 4 Center of Innovation for Complex Chronic Healthcare (CINCCH), Edward Hines, Jr. VA Hospital, Hines, IL USA; 5 Department of Surgery and Renal Transplant, Edward Hines Jr. Veterans Administration Hospital, Hines, IL USA; 6 Department of Surgery and Renal Transplant, Loyola University Chicago, Maywood, IL USA; 7 Department of Public Health Sciences, Parkinson School of Health Science and Public Health, Loyola University Chicago, Maywood, IL USA; 8 Division of Geriatrics and Palliative care, University of Chicago, Chicago, IL USA

**Keywords:** Frailty, Hemodialysis, Renal transplantation, Kidney Disease

## Abstract

**Background:**

Frailty increases risk of morbidity and mortality in hemodialysis patients. Frailty assessments could trigger risk reduction interventions if broadly adopted in clinical practice. We aimed to assess the clinical feasibility of frailty assessment among Veteran hemodialysis patients.

**Methods:**

Hemodialysis patients’ ≥50 years were recruited from a single dialysis unit between 9/1/2021 and 3/31/2022.Patients who consented underwent a frailty phenotype assessment by clinical staff. Five criteria were assessed: unintentional weight loss, low grip strength, self-reported exhaustion, slow gait speed, and low physical activity. Participants were classified as frail (3–5 points), pre-frail (1–2 points) or non-frail (0 points). Feasibility was determined by the number of eligible participants completing the assessment.

**Results:**

Among 82 unique dialysis patients, 45 (52%) completed the assessment, 13 (16%) refused, 18 (23%) were not offered the assessment due to death, transfers, or switch to transplant or peritoneal dialysis, and 6 patients were excluded because they did not meet mobility criteria. Among assessed patients, 40(88%) patients were identified as pre-frail (46.6%) or frail (42.2%). Low grip strength was most common (90%). Those who refused were more likely to have peripheral vascular disease (*p* = 0.001), low albumin (*p* = 0.0187), low sodium (*p* = 0.0422), and ineligible for kidney transplant (*p* = 0.005).

**Conclusions:**

Just over half of eligible hemodialysis patients completed the frailty assessment suggesting difficulty with broad clinical adoption expectations. Among those assessed, frailty and pre-frailty prevalence was high. Given patients who were not tested were clinically high risk, our reported prevalence likely underestimates true frailty prevalence. Providing frailty reduction interventions to all hemodialysis patients could have high impact for this group.

## Introduction

Frailty is a medical syndrome characterized by reduced physiologic function and strength resulting in increased vulnerability to dependence on others and death [1]. Aging and chronic conditions such as end stage kidney disease (ESKD) requiring dialysis contribute to frailty through various mechanisms including sarcopenia, oxidative stress, chronic inflammation, and alterations in hormonal balance [2–5]. The estimated prevalence of frailty in community dwelling adults is 10% [6], whereas the prevalence of frailty in older adults with ESKD ranges from 30 to 60% [7–9]. ESKD prevalence has risen among older individuals ages 65 and older in the last decade (2009 to 2019), with a 15% increase among individuals aged ≥ 75 years and 12% increase among individuals aged 65–74 years [10]. Frailty prevalence is, therefore, also expected to increase.

In patients with ESKD, frailty has been associated with increased mortality, hospitalizations (twice as likely), falls (three times as likely), fractures, cognitive decline, and vascular access failure [11–14]. Frail individuals with ESKD have worse health-related quality of life over time [15]. Being frail lowers the propensity of receiving a kidney transplant among dialysis patients. Frailty reduction interventions have the potential to improve functional outcomes and quality of life, and to improve equitable access to kidney transplant [16–18]. A review of eight RCTs including 290 patients with ESKD receiving dialysis showed strength training was associated with important clinical outcomes including improved muscle strength and improved self-rated physical health and function [19]. Several medical organizations recommend routine frailty assessment including the International society of Geriatric Oncology for older cancer patients [20], the American College of Surgeons National Surgical Quality Improvement Program and the American Geriatrics Society for older patients undergoing surgical procedures [21], the American Diabetes Associations for older diabetics [22], and the American College of Cardiology/ American Heart Association Joint Committee on Clinical practice Guidelines for patients with valvular heart disease [23]. Formal frailty assessment for management of older patients with chronic kidney disease stage 3 b or higher is also suggested by the European Renal Best Practice Working Group clinical practice guideline. No similar guidance is available from national nephrology societies in the United States at the current time. ​.

Screening for frailty among ESKD patients would help identify those who may benefit from frailty reduction initiatives; however, frailty assessment has not become part of routine ESKD care, yet. There are many provider and patient challenges to frailty assessment feasibility in this specific population. Among provider challenges are lack of guidance on when and how to assess frailty, time restrictions, lack of frailty assessment training, and lack of reimbursement [24, 25]. Among patient challenges are fatigue due to fluid and electrolyte shifts, reluctance to attend extra patient visits, and additional time needed for frailty assessment during an existing visit or already lengthy dialysis session [26].

The primary objective of the current study was to determine the feasibility of assessing frailty during the hemodialysis visit at a Veteran Affairs (VA) Hospital dialysis unit. Our secondary objective was to describe the prevalence of frailty and each frailty criteria in a hemodialysis patient sample. We conducted our analyses in patients 50 years and older receiving hemodialysis at a single-center dialysis unit. Frailty was assessed using the Johns Hopkins frailty calculator which is an adaptation of the Fried frailty phenotype [6]. Feasibility was assessed by the proportion of patients who were eligible for frailty assessment and who consented to complete frailty testing. We used well-established definitions of feasibility from prior implementation science literature, particularly Proctor et al’s 2011 paper, which defined feasibility as the “extent to which a new treatment, or an innovation, can be successfully used or carried out within a given agency or setting [27].”

## Study design and methods

### Study design

This is a cross-sectional descriptive study at a single-site dialysis unit. Frailty assessments were collected in-person and demographic and health condition data were collected from the electronic health record (EHR) review.

### Participants

Eligible patients 50 years and older receiving hemodialysis at Edward Hines VA dialysis unit were recruited to participate in frailty assessment from 9/1/2021 through 3/31/2022 during their dialysis session. The focus of the study was age 50 and older patients because of higher prevalence of frailty among this aging dialysis population [10]. Patients were not eligible in this study if they met any of the following exclusion criteria identified from the EHR: experienced a cardiovascular event such as stroke or myocardial infarction in the last 6 months, fracture in the last 3 months, or had any of the following conditions including advanced dementia, paraplegia, uncontrolled arrythmias, dissecting aortic aneurysm, acute endo/pericarditis, acute thromboembolism, acute or severe heart or respiratory failure, uncontrolled hypertension (> 180/100 mm/Hg,) or active infections affecting one’s general health condition. Due to these exclusion criteria, there were 6 patients that were excluded due to paraplegia/ amputation/ bed bound status. Additional 18 patients were not offered frailty assessment because they expired during screening period (n = 7), transferred to another unit (n = 7), relocated to another city (n = 2), transplanted, or switched to peritoneal dialysis (n = 2).

### Procedure

Patients who consented to frailty assessment were scheduled for the evaluation either before or after their next dialysis visit based on patient preference. For patients during the early morning dialysis shift (between 5 am to 10 am), frailty assessments were conducted after dialysis (n = 23). For patients during the mid-morning dialysis shift (between 1030 am to 330 pm), frailty assessments were conducted before their dialysis (n = 22). The dialysis center dietician and exercise physiologist with no previous background in frailty were trained to administer the frailty assessments and were initially observed by the PI to assure accurate measurement. Coordinating frailty assessments required additional study personnel and clinic staff including the unit’s social worker and medical assistant time because patients needed to be reminded to either come early before dialysis or stay later after dialysis for the assessments and/or help them rearrange transportation to the unit since a large portion of these patients rely on the VA or family members for transportation needs. Throughout study period, the study team met regularly to discuss facilitators and challenges to conducting the frailty assessments as well as possible strategies to address these challenges.

### Frailty assessment

Frailty was assessed using the Johns Hopkins frailty calculator which adapted the Fried frailty phenotype [6, 28, 29]. Patients were assigned a score from 0 to 5. One point was assigned for the presence of each of the following five criteria: recent unintentional weight loss, low grip strength, exhaustion, slow walk speed, and low physical activity. Unintentional weight loss was assessed using measured weight from the EHR over the past year and validated by checking with the patient if the weight loss was intentional or unintentional. Grip strength was assessed using Jamar dynamometer on the dominant hand, (maximal score of the 3 measurements is used). Exhaustion was self-reported using questions related to tiredness, weakness, and energy levels over the past month. Walk speed was assessed using a 4-meter usual walking test, physical activity was assessed using the self-reported 6-Item Minnesota Leisure Time Physical Activity Questionnaire which included 6 activities (walking, doing strenuous household chores, strenuous outdoor chores, dancing, bowling, and exercise). Patients were classified as frail if they demonstrated three to five of the frailty criteria and pre-frail if they demonstrated one or two of the frailty criteria.

### Covariates

Demographics and clinical characteristics potentially associated with frailty were extracted from the EHR. These included age, gender, Body Mass Index (BMI), co-morbidities [diabetes, hypertension, cerebrovascular accident (CVA), obstructive sleep apnea (OSA), obesity, peripheral vascular disease (PVD), congestive heart failure (CHF) or coronary artery disease (CAD)], medications, type of vascular access [arteriovenous fistula (AVF), arteriovenous graft (AVG) or catheter], dialysis vintage, Charlson Comorbidity Index, selected lab results [blood urea nitrogen, urea reduction ratio, sodium, potassium, phosphorus, parathyroid hormone, calcium, albumin, hemoglobin], and current transplant waitlist status [not a candidate, workup in progress, listed for transplant].

### Statistical analysis

The proportion of ineligible patients, eligible patients who did not consent, and eligible patients who consented were reported. Bivariate analysis was used to compare the demographic and clinical characteristics of patients who were offered frailty assessment by assessment completion status. Univariate analysis was used to describe and plot frailty prevalence in the participants. Bivariate analysis was used to compare the demographic and clinical characteristics of patients across frailty subgroups: non-frail, pre-frail, and frail. We used the t-test and ANOVA statistics for continuous dependent variables, Wilcoxon rank-sum and Kruskal-Wallis tests for nonparametric dependent variables that are non-normally distributed, and the Chi-2 test and Fisher exact test (when expected cell value was < 5) for categorical variables. The kidney transplant status variable was missing for two patients (because one patient died and the other transferred to another unit). Therefore, we excluded these missing observations when conducting the bivariate analysis for this variable. All analysis was conducted in Stata 17 (StataCorp, College Station, TX). All *p*-values were for two-sided tests and statistical significance was defined as alpha = 0.05.

## Results

**Fig. 1:**
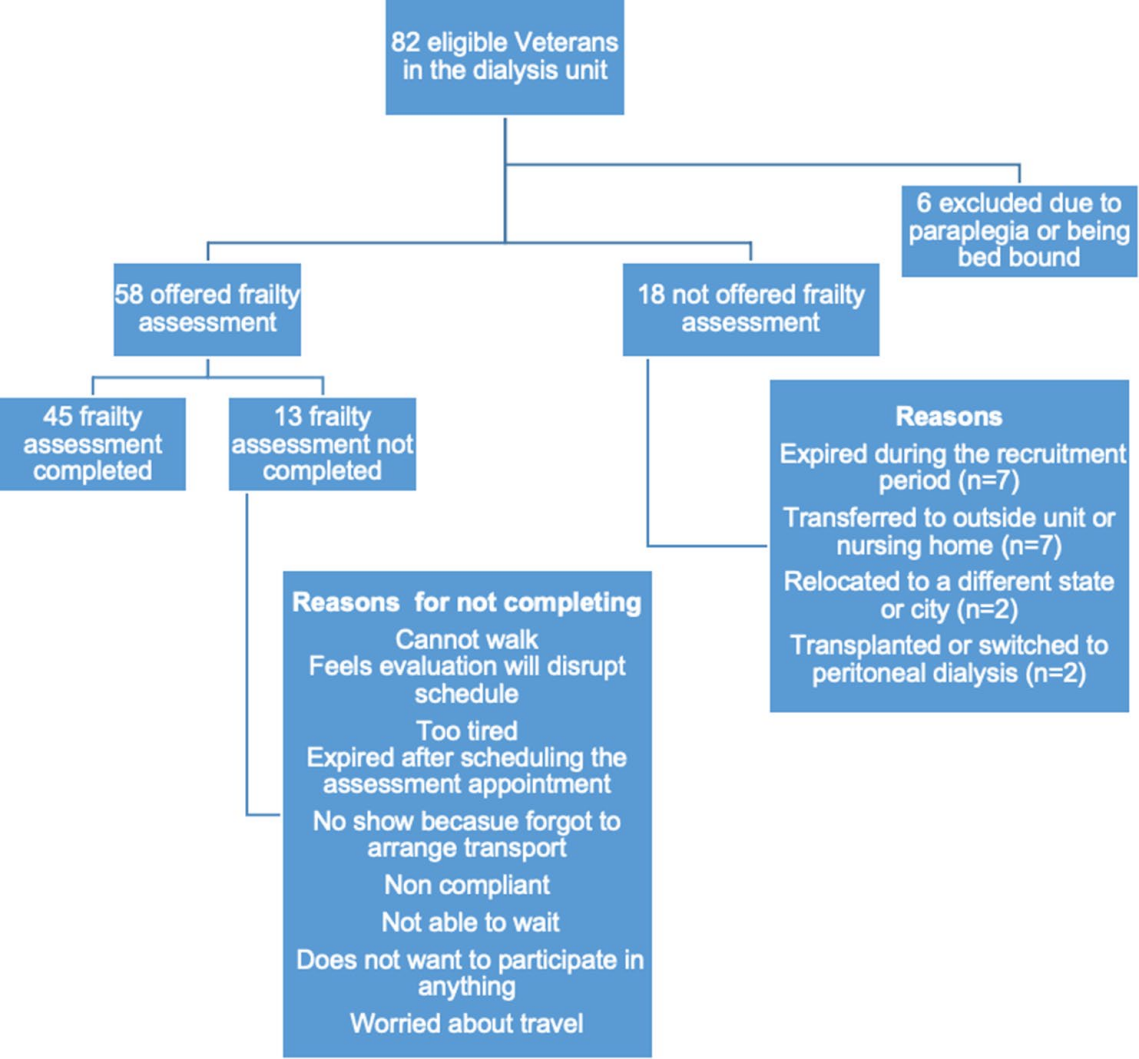
Study Enrollment diagram

*Frailty assessment feasibility (shown in* Fig. 1). Among 82 unique patients in the dialysis unit, 76 were eligible for frailty assessment during the study period (9/1/2021- 3/31/2022). Six patients were not eligible for this study because they were either paraplegic, bed bound and/or amputee. Of eligible patients, frailty assessment was not offered to 18 patients due to the following reasons: expired during recruitment time frame (n = 7), transferred to an outside unit or nursing home (n = 7), relocated to another city/state (n = 2), transplanted/ modality switch to peritoneal dialysis (n = 2). Remaining 58 patients were offered frailty assessment up to three times during separate hemodialysis sessions. Of these patients, 45 completed the assessment and 13 patients refused or were unable to complete the assessment. Among the 13 patients who refused frailty assessment, reasons included too tired after dialysis, did not want to rearrange transportation to accommodate assessment, did not want to wait for assessment, noncompliance with dialysis treatments hence did not show for assessment. Among the 45 patient participants who completed assessment, the frailty assessment took on average between 5 and 15 min.

Table 1 summarizes the participant characteristics among those offered a frailty assessment comparing those who completed the assessment versus those who did not. Data showed that those who declined frailty assessments were significantly more likely to have peripheral vascular disease (PVD) (61.5% vs. 13.3%, *p* = 0.001), low albumin (3.4 g/dL vs. 3.8 g/dL, *p* = 0.0187), low sodium (128.4 mmol/L vs. 136.8 mmol/L, *p* = 0.0422), use of vasodilator medications (53.8% vs. 20.0%, *p* = 0.031) and been declined for kidney transplant listing (100% vs. 53.5%, *p* = 0.005).

**Table 1:** Dialysis patient characteristics who were offered frailty assessment by assessment completion (n = 58)

Characteristics	Did not complete or refuse frailty assessment (n = 13)	Completed frailty assessment (n = 45)	*p*-value	Total
Dialysis shift, early morning shift	7 (53.8%)	23 (51.1%)	p = 0.862	30 (51.7%)
Mid-morning shift	6 (46.2%)	22 (48.9%)		28 (48.3%)
Testing day, MWF	6 (46.2%)	24 (53.3%)	p = 0.648	30 (51.7%)
TTS	7 (53.8%)	21 (46.7%)		28 (48.3%)
Age, mean (s.d.)	68.2 (7.0)	69.9 (10.2)	p = 0.5850	69.5 (9.5)
Gender, Male	13 (100%)	44 (97.8%)	p = 1.000	57 (98.3%)
Race/ethnicity, NH white	4 (30.8%)	18 (40.0%)	p = 0.845	22 (37.9%)
NH black	8 (61.5%)	22 (48.9%)		30 (51.7%)
NH Asian	0	1 (2.2%)		1 (1.7%)
Hispanic	1 (7.7%)	4 (8.9%)		5 (8.6%)
BMI, mean (s.d.)	30.1 (9.4)	29.1 (7.0)	p = 0.6657	29.3 (7.0)
Diabetes	11 (84.6%)	34 (75.6%)	p = 0.711	45 (77.6%)
Hypertension	12 (92.3%)	39 (86.7%)	p = 1.000	51 (87.9%)
Cerebrovascular accident (CVA)	2 (15.4%)	5 (11.1%)	p = 0.648	7 (12.1%)
Obstructive sleep apnea (OSA)	5 (38.5%)	17 (37.8%)	p = 1.000	22 (37.9%)
Obesity	6 (46.1%)	14 (31.1%)	p = 0.339	20 (34.5%)
Peripheral vascular disease (PVD)	8 (61.5%)	6 (13.3%)	**p = 0.001**	14 (24.1%)
Congestive heart failure or coronary artery disease	8 (61.5%)	19 (42.2%)	p = 0.219	27 (46.5%)
Vascular access, catheter	3 (23.1%)	5 (11.1%)	p = 0.385	8 (13.8%)
Arteriovenous graft	4 (30.8%)	10 (22.2%)		14 (24.1%)
Arteriovenous fistula	6 (46.2%)	30 (66.7%)		36 (62.1%)
Access failure, yes	5 (38.5%)	16 (35.6%)	p = 1.000	21 (36.2%)
Dialysis vintage, mean (s.d.)	5.9 (3.3)	4.1 (3.2)	p = 0.07	4.5 (3.3)
KT waitlist status, not a candidate	13 (100%)	23 (53.5%)	**p = 0.005**	36 (64.3%)
Work up in progress	0	17 (39.5%)		17 (30.4%)
Listed	0	3 (7.0%)		3 (5.4%)
Urea Reduction Ratio (> 67%), mean (s.d.)	73.2% (4.4)	74.0% (4.5)	p = 0.55	73.8% (4.5)
KT/V ( > = 1.2), mean (s.d.)	1.5 (0.2)	1.5 (0.2)	p = 0.8091	1.5 (0.2)
Potassium (3.5–4.7 mmol/L), mean (s.d.)	4.5 (0.6)	4.3 (0.5)	p = 0.3205	4.3 (0.5)
Sodium (135–145 mmol/L), mean (s.d.)	128.4 (27.3)	136.8 (2.8)	**p = 0.0422**	134.9 (13.2)
Phosphorous (3.5–5.5 XX), mean (s.d.)	4.9 (1.3)	4.6 (1.0)	p = 0.3416	4.7 (1.0)
PTH (< 600 pg/mL), mean (s.d.)	902.1 (1139.0)	640.0 (398.2)	p = 0.1948	698.7 (638.5)
Calcium (8.5–10.3 mg/dL), mean (s.d.)	8.7 (0.8)	8.8 (0.5)	p = 0.5458	58 (0.6)
Albumin (3.5–4.2 g/dL), mean (s.d.)	3.4 (0.8)	3.8 (0.6)	**p = 0.0187**	3.7 (0.7)
Hemoglobin (9–11 g/dl), mean (s.d.)	10.2 (1.0)	10.3 (1.2)	p = 0.8005	10.3 (1.1)
Charlson comorbidity index, median (IQR)	8 (7–9)	7 (5–8)	p = 0.2216	7.3 (6–9)
Estimated 10 years survival, 0%	11 (84.6%)	30 (66.7%)	p = 0.106	41 (70.7%)
2%	2 (15.4%)	2 (4.4%)		4 (6.9%)
21%	0	11 (24.4%)		11 (19.0%)
53%	0	2 (4.4%)		2 (3.4%)
Pill count, mean (s.d.)	18.3 (10.2)	19.3 (8.9%)	p = 0.7898	19.1 (9.1)
Life sustaining treatment, None	1 (7.7%)	12 (26.7%)	p = 0.407	13 (22.4%)
DNR and/or DNI	3 (23.1%)	7 (15.6%)		10 (17.2%)
Full code	9 (69.2%)	26 (57.8%)		35 (60.3%)
ACE or ARB, yes	1 (7.7%)	5 (11.1%)	p = 0.594	6 (10.3%)
CCB, yes	7 (53.8%)	15 (33.3%)	p = 0.208	22 (37.9%)
Beta blockers, yes	9 (69.2%)	22 48.9%)	P = 0.195	31 (53.4%)
Diuretics, yes	1 (7.7%)	13 (28.9%)	P = 0.155	14 (24.1%)
Alpha blockers, yes	0	5 (11.1%)	p = 0.267	5 (8.6%)
Alpha-2 agonists, yes	0	1 (2.2%)	p = 1.000	1 (1.72%)
Vasodilators, yes	7 (53.8%)	9 (20.0%)	**p = 0.031**	16 (27.6%)
Midodrine for hypotension during dialysis, yes	3 (23.1%)	12 (26.7%)	p = 1.000	15 (25.9%)
ESA for anemia management, yes	11 (84.6%)	35 (77.8%)	p = 0.716	46 (79.3%)
Phosphate binders, yes	9 (69.2%)	28 (62.2%)	p = 0.751	37 (63.8%)

NH, Non-Hispanic; MWF, Mon/Wed/Fri; TTS, Tues/Thurs/Sat; BMI, Body Mass Index; KT, kidney transplant; IQR, interquartile range; DNR, do not resuscitate; DNI, do not intubate; PTH, parathyroid hormone; ACE, angiotensin-converting enzyme; ARB, angiotensin II receptor blockers; CCB, calcium channel blocker; ESA, Erythropoietin stimulating agents

**Fig. 2:**
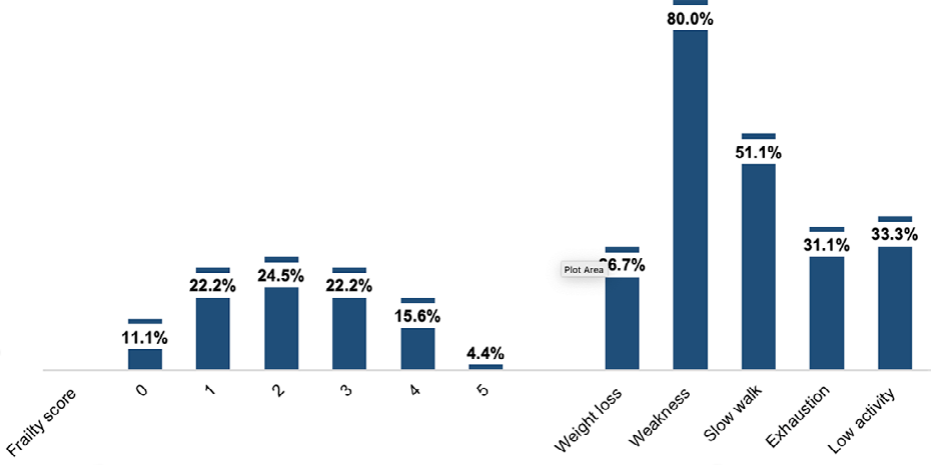
Frailty prevalence among hemodialysis patients (n = 45)

**Fig. 3:**
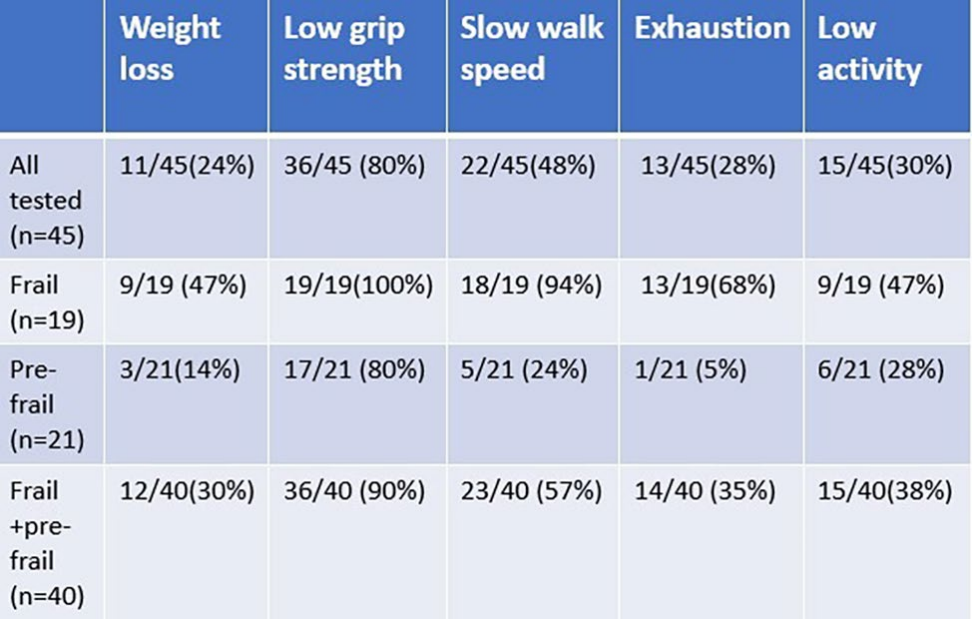
Frequency of individual frailty indicators

*Frailty prevalence*. Among the 45 hemodialysis patients who completed the frailty assessment, there were 5 non-frail (11%), 21 pre-frail (47%), and 19 (42%) frail patients (shown in Fig. 2). Among the 40 patients who were pre-frail or frail, weak grip strength was the most common frailty criteria (90%), followed by slow gait (57%), low physical activity (38%), exhaustion (35%), and weight loss (30%) (shown in Fig. 3). There was no association between dialysis shift (early morning vs. mid-morning) and frailty score (0, 1–2, and 3–5) (Fisher’s exact p = 0.411).

Table 2 summarizes the patient characteristics across frailty subgroups. The mean age of participants was 69.9 years, with 31 patients (68.9%) older than 65 years. Participants were predominantly male (97.8%) and almost 50% were black. Prevalence of diabetes (75%) and hypertension (85%) were high. About 67% patients had an arteriovenous fistula (AVF), 22% had arteriovenous graft (AVG), and a smaller subset of patients had a catheter as dialysis access. About 35% of patients had previous failed access and almost 40% of patients were undergoing transplant workup. The average age was highest among frail Veterans (mean = 71.7 years) followed by pre-frail Veterans (mean = 70.4). Non-frail Veterans were the youngest, typically, with a mean age of 60.4. Mean dialysis vintage time was longer for pre-frail and frail, 4.6 and 4 years, respectively, compared to 1.9 years for non-frail populations. The burden of comorbidities was higher among pre-frail and frail group. The CCI was 5 (IQR 5–5) for the non-frail group compared to 8 (IQR 6–9) in the pre frail and frail groups.

**Table 2:** Hemodialysis patient characteristics by frailty assessment score (n = 45)

Characteristics	Frailty score 0 (n = 5)	Frailty score 1–2 (n = 21)	Frailty score 3–5 (n = 19)	*p*-value	Total
Age, mean (s.d.)	60.4 (8.4)	70.5 (10.2)	71.7 (9.7)	p = 0.0776	69.9 (10.2)
Gender, Male	5 (100%)	20 (95.2%)	10 (100%)	p = 1.000	44 (97.8%)
Race/ethnicity, NH white	1 (20.0%)	9 (42.9%)	8 (42.1%)	p = 0.185	18 (40.0%)
NH black	4 (80.0%)	7 (33.3%)	11 (57.9%)		22 (48.9%)
NH Asian	0	1 (4.8%)	0		1 (2.2%)
Hispanic	0	4 (10.0%)	0		4 (8.9%)
BMI, mean (s.d.)	33.3 (8.4)	29.9 (6.8)	27.1 (4.1)	p = 0.0901	29.1 (6.2)
Diabetes	3 (60.0%)	16 (76.2%)	15 (78.9%)	p = 0.700	34 (75.6%)
Hypertension	5 (100%)	19 (90.5%)	15 (79.0%)	p = 0.452	39 (86.7%)
Cerebrovascular accident (CVA)	0	3 (14.3%)	2 (10.5%)	p = 1.000	5 (11.1%)
Obstructive sleep apnea (OSA)	2 (40.0%)	9 (42.9%)	6 (31.6%)	p = 0.827	17 (37.8%)
Obesity	1 (20.0%)	9 (42.9%)	4 (21.0%)	p = 0.356	14 (31.1%)
Peripheral vascular disease (PVD)	0	3 (14.3%)	3 (15.8%)	p = 1.000	6 (13.3%)
Congestive heart failure or coronary artery disease	0	8 (38.1%)	11 (57.9%)	p = 0.071	19 (42.2%)
Vascular access, catheter	3 (60.0%)	1 (4.8%)	1 (5.3%)	**p = 0.015**	5 (11.1%)
Arteriovenous graft	1 (20.0%)	3 (14.3%)	6 (31.6%)		10 (22.2%)
Arteriovenous fistula	1 (20.0%)	17 (81.0%)	12 (63.2%)		30 (66.7%)
Access failure, yes	3 (60.0%)	9 (42.9%)	4(21.0%)	p = 0.180	16 (35.6%)
Dialysis vintage, mean (s.d.)	1.9 (1.5)	4.6 (3.6)	4.0 (3.0)	p = 0.2318	4.0 (3.2)
KT waitlist status, not a candidate	0	12 (57.1%)	11 (61.1%)	p = 0.144	23 (53.5%)
Work up in progress	3 (75.0%)	8 (38.1%)	6 (33.3%)		17 (39.5%)
Listed	1 (25.0%)	1 (4.8%)	1 (5.6%)		3 (7.0%)
Urea Reduction Ratio (> 67%), mean (s.d.)	70.5 (4.3)	75.2 (4.7)	73.6 (4.0)	p = 0.1030	74.0 (4.5)
KT/V ( > = 1.2), mean (s.d.)	1.3 (0.1)	1.5 (0.2)	1.4 (0.2)	p = 0.0783	1.5 (0.2)
Potassium (3.5–4.7 mmol/L), mean (s.d.)	4.2 (0.2)	4.2 (0.4)	4.4 (0.6)	p = 0.3754	4.3 (0.5)
Sodium (135–145 mmol/dL), mean (s.d.)	139.0 (1.2)	136.8 (3.0)	136.2 (2.6)	p = 0.1328	136.8 (2.8)
Phosphorous (3.5–5.5 mg/dL), mean (s.d.)	4.8 (1.1)	4.5 (0.7)	4.7 (1.2)	p = 0.8175	4.6 (1.0)
PTH (< 600 pg/dL), mean (s.d.)	668.8 (204.9)	577.2 (405.6)	701.8 (432.0)	p = 0.6154	640.0 (398.2)
Calcium (8.5–10.3 mg/dL), mean (s.d.)	8.7 (0.4)	8.7 (0.5)	9.0 (0.5)	p = 0.0624	8.8 (0.5)
Albumin (3.5–4.2 g/dL), mean (s.d.)	4.2 (0.3)	3.8 (0.4)	3.8 (0.7)	p = 0.3318	3.8 (0.6)
Hemoglobin (9–11 g/dl), mean (s.d.)	10.7 (0.7)	10.5 (1.0)	9.9 (1.4)	p = 0.2150	10.3 (1.2)
Charleston comorbidity index, median (IQR)	5 (5–5)	8 (6–9)	8 (6–8)	p = 0.1824	7 (5–8)
Estimated 10 years survival, 0%	1 (20.0%)	15 (71.4%)	14 (73.7%)	p = 0.099	30 (66.7%)
2%	0	1 (4.8%)	1 (5.3%)		2 (4.4%)
21%	3 (60.0%)	5 (23.8%)	3 (15.8%)		11 (24.4%)
53%	1 (20.0%)	0	1 (5.3%)		2 (4.4%)
Pill count, mean (s.d.)	16.2 (6.0)	17.7 (10.9)	21.9 (6.4)	p = 0.2582	19.3 (8.9)
Life sustaining treatment, None	3 (60.0%)	7 (33.3%)	2 (10.5%)	p = 0.133	12 (26.7%)
DNR and/or DNI	0	2 (9.5%)	5 (26.3%)		7 (15.6%)
Full code	2 (40.0%)	12 (57.1%)	12 (63.2%)		26 (57.8%)
ACE or ARB, yes	0	1 (4.8%)	4 (21.0%)	p = 0.221	5 (11.1%)
CCB, yes	4 (80.0%)	6 (28.6%)	5 (26.3%)	p = 0.084	15 (33.3%)
Beta blockers, yes	3 (60.0%)	11 (52.4%)	8 (42.1%)	p = 0.697	22 (48.9%)
Diuretics, yes	1 (20.0%)	7 (33.3%)	5 (26.3%)	p = 0.899	13 (28.9%)
Alpha blockers, yes	0	4 (19.0%)	1 (5.3%)	p = 0.397	5 (11.1%)
Alpha-2 agonists, yes	0	1 (4.8%)	0	p = 1.000	1 (2.2%)
Vasodilators, yes	2 (40.0%)	3 (14.3%)	4 (21.0%)	p = 0.378	9 (20.0%)
Midodrine for hypotension during dialysis, yes	1 (20.0%)	3 (14.3%)	8 (42.1%)	p = 0.124	12 (26.7%)
ESA for anemia management, yes	2 (40.0%)	17 (81.0%)	16 (84.2%)	p = 0.138	35 (77.8%)
Phosphate binders, yes	4 (80.0%)	12 (57.1%)	12 (63.2%)	p = 0.634	28 (62.2%)

## Discussion

The current analysis is among the first to assess the extent to which frailty phenotype can be assessed during clinical care among patients on hemodialysis and contributes to the frailty phenotype prevalence uniquely in VA patients on hemodialysis. Our team was only able to successfully measure the frailty phenotype in a little over half of all eligible hemodialysis patients over the recruitment period. Among hemodialysis patients who were assessed, our study shows a very high prevalence of pre-frailty and frailty (88%). Our reported frailty prevalence likely underestimates true frailty prevalence in this group given the high-risk health characteristics of patients who were not screened.

There are many patient, provider, and health systems level barriers to implement frailty assessment in hemodialysis patients. Some of these barriers may not be unique to ESKD patients, but some are. ESKD patients need multiple visits each week to dialysis unit, as a result most dialysis patients may not wish to come for additional visits to the health center to perform frailty assessment. To alleviate this barrier, we performed assessments during their dialysis session visit. Additional staff time was needed to schedule these assessments during clinical care. The social worker and medical support assistant also helped adjust patient transportation schedules to accommodate the extra time required for the assessment, albeit short, if not properly planned, may disrupt busy dialysis unit schedules. Clinical providers and staff may not have frailty administration training which poses another hurdle. Our team members were able to self-train using the John Hopkins frailty calculator website. Additionally, cost of some the new equipment needed for the assessment (example dynamometer) should be accounted for (~ $250–400). Dialysis patients are weighed before and after dialysis treatments, hence data for height and weight can be used from electronic records and verified with the patient. While we did not assess frailty in our paraplegic and bedbound patients due to safety concerns in the area used for these assessments, exhaustion, weight loss and grip strength could be assessed in patients who are bedbound. For patients who are bedbound as a result of progressive chronic illness, the gait speed and physical activities could be appropriately classified as “low.” Little research is available to guide the adaptation of the frailty criteria in patients who are paraplegic but otherwise healthy.

Identifying dedicated time to conduct these assessments by busy dialysis unit staff remains a major challenge for implementing routine frailty assessment. While frailty evaluations were generally brief (5–15 min), coordinating patient availability (same day as dialysis) with the availability of the frailty assessment staff was challenging and led to some delays in scheduling these assessments. The dialysis unit is a highly regulated environment with protocols and reporting requirements. Dialysis staff have assigned tasks that they must perform to provide high quality, safe dialysis care. One of the dialysis team members must be identified, be trained and provided dedicated time to perform these evaluations.

A multitude of quality parameters and outcomes are monitored but currently, frailty is not one of them. If frailty evaluations were included in this list, existing personnel could conduct frailty testing with adequate administrative support.

We found a significant number (n = 13) of hemodialysis patients who were eligible for assessment but declined it. We found that those who declined frailty assessment were more likely to have peripheral vascular disease (61.5% versus 13.3%, *p* = 0.001) and were previously deemed not to be transplant candidates. These findings suggest that there may be lack of motivation, obvious incentive, or knowledge of benefits of frailty assessment at the patient level that must be explored and addressed to optimize implementation [30–32].

Based on a meta-analysis of frailty in dialysis studies, prevalence of frailty ranged from 29.6 to 81.5% among all studies [33]. Three of the 7 larger U.S. studies included in this meta-analysis used the Fried’s frailty score and reported a prevalence of frailty (score 3 or higher) as 61/146 (41.8%) McAdams-DeMarco 2013 [33], 177/370 (47.8%) Fitzpatrick et al. 2019 NDT [34], 230/727 (31.6%) Johansen et al. 2019 [35]. Our study shows 19/45 (42%) of our veterans who were screened had a frailty score of 3 or higher using similar criteria. Fitzpatrick et al. study had slow gait speed as the predominant criteria finding in 62%, followed by low physical activity in 53% and unintentional weight loss and grip strength in 50% of their participants. Johansen et al. study showed 54.5% had weak grip strength, 28.2% had slow gait speed and 40.9% had low physical activity. Weak grip strength was the most prevalent criteria in our study, being present among 100% of frail and 80% of pre-frail population. Several factors can play a role in weak grip strength among hemodialysis patients including restrictions imposed on upper extremity strengthening exercises to avoid damaging hemodialysis access. In the absence of specific guidance about how much weight dialysis patients with arteriovenous fistula (AVF) can safely lift, most AVF patients are advised to avoid carrying heavy objects in the arm with AVF. One recent study tried to address this issue. In this study of 86 dialysis patients, patients were randomized to exercising with 6-lb dumbbells (dumbbell group) or squeezable rubber balls (handgrip group). They found no differences between the two groups regarding AVF patency and complications including aneurysm, puncture site hematoma, hemorrhage, or cardiac failure, suggesting safety of moderate weight exercise in this population [36]. Dialysis access can fail, and patients may require additional surgeries for creation of new dialysis access propagating the cycle of not exercising or lifting with the arm with dialysis access. Lack of guidance about the amount of exercise or lifting that can be performed with dialysis access arm likely exacerbates sarcopenia of the arm and hence the weak grip strength that needs to be further addressed in larger studies. Accordingly targeted interventions focusing on improving grip strength in dialysis patients are needed.

Slow walking speed was identified in 90% of our frail Veterans. While we did not find statistically significant difference in PVD and frailty in our population, PVD has been previously associated with slow walking speed. A large study of frailty in a dialysis population showed 78% of their sample had slow walking speed. In adjusted analyses, PVD and cardiac disease were associated with higher odds of frailty [37].

### Study limitations

Because of the small number of patients per group in some of the bivariate analysis, especially the associations between patient characteristics and frailty score, our associations did not reach statistical significance. More robust associations could be observed with a larger sample size and number of patients per frailty score group. Also, since our sample size was small, especially for patients with frailty score 0, some of our observed associations may be due to chance. Our frailty estimates may be an underestimation in this population as those not offered frailty assessment or who were not able to complete the frailty assessment may have had a higher prevalence of frailty. It is possible that restricting our sample with exclusion criteria based on recent medical events may have caused our feasibility to be better than in real practice. We recruited VA patients which included a higher proportion of African- American and predominantly male patients; therefore, our findings may not be generalizable to other hemodialysis populations. In this pilot study, we described barriers and potential strategies based on the study teams’ (that included clinicians, exercise physiologist, and researchers) experiences implementing the frailty assessment in the dialysis unit. However, a qualitative approach using in-depth interviews and focus groups can identify patient and provider barriers and facilitators, provider readiness, and perspectives about frailty assessment in the dialysis unit and frailty reduction interventions for this population that can inform clinical practice regarding frailty assessment in dialysis units.

## Conclusion

Frailty is an essential evaluation in older adults with ESRD and will become increasingly important as the older ESRD population increases. Frailty reduction has the potential to improve access to transplantation and outcomes for the dialysis population and to reduce cost of care. We outlined important feasibility challenges to overcome for broad, routine testing in future implementation studies. Our study suggests that frailty is highly prevalent in older hemodialysis patients, rates that are likely underestimated due to assessment challenges. Weak grip strength is a predominant, targetable frailty feature in nearly all hemodialysis patients with frailty. Efforts to expand implementation of frailty testing systemwide in the VA must be reinforced at the policy level and leverage dedicated, multidisciplinary teams while accounting for the care coordination needs of this complex patient population.

## Data Availability

All data generated or analyzed during this study are included in this article. Further enquiries can be directed to the corresponding author.
